# The Taxon Hypothesis Paradigm—On the Unambiguous Detection and Communication of Taxa

**DOI:** 10.3390/microorganisms8121910

**Published:** 2020-11-30

**Authors:** Urmas Kõljalg, Henrik R. Nilsson, Dmitry Schigel, Leho Tedersoo, Karl-Henrik Larsson, Tom W. May, Andy F. S. Taylor, Thomas Stjernegaard Jeppesen, Tobias Guldberg Frøslev, Björn D. Lindahl, Kadri Põldmaa, Irja Saar, Ave Suija, Anton Savchenko, Iryna Yatsiuk, Kristjan Adojaan, Filipp Ivanov, Timo Piirmann, Raivo Pöhönen, Allan Zirk, Kessy Abarenkov

**Affiliations:** 1 Natural History Museum, University of Tartu, 14a Ravila, 50411 Tartu, Estonia; kadri.poldmaa@ut.ee (K.P.); ave.suija@ut.ee (A.S.); kristjan.adojaan@ut.ee (K.A.); filipp.ivanov@ut.ee (F.I.); timo.piirmann@ut.ee (T.P.); raivo.pohonen@ut.ee (R.P.); allan.zirk@ut.ee (A.Z.); kessy.abarenkov@ut.ee (K.A.); 2 Institute of Ecology and Earth Sciences, University of Tartu, 14a Ravila, 50411 Tartu, Estonia; leho.tedersoo@ut.ee (L.T.); irja.saar@ut.ee (I.S.); anton.savchenko@ut.ee (A.S.); iryna.yatsiuk@ut.ee (I.Y.); 3 Department of Biological and Environmental Sciences, Gothenburg Global Biodiversity Centre, University of Gothenburg, Box 461, 405 30 Göteborg, Sweden; henrik.nilsson@bioenv.gu.se (H.R.N.); k.h.larsson@nhm.uio.no (K.-H.L.); 4 Global Biodiversity Information Facility, 2100 Copenhagen, Denmark; dschigel@gbif.org (D.S.); tsjeppesen@gbif.org (T.S.J.); 5 Royal Botanic Gardens Victoria, Birdwood Ave, Melbourne, Victoria 3004, Australia; tom.may@rbg.vic.gov.au; 6 The James Hutton Institute, Craigiebuckler, Aberdeen AB15 8QH, UK; Andy.Taylor@hutton.ac.uk; 7 Institute of Biological and Environmental Sciences, University of Aberdeen, Cruickshank Building, St Machar Drive, Aberdeen AB24 3UU, UK; 8 GLOBE Institute, University of Copenhagen, Øster Voldgade 5-7, 1350 København, Denmark; tobiasgf@sund.ku.dk; 9 Systematic Biology, Evolutionary Biology Centre, Uppsala University, Norbyvägen 18D, 75236 Uppsala, Sweden; bjorn.lindahl@slu.se

**Keywords:** microbial species, taxonomy, DNA taxonomy, biodiversity informatics, discovery of species, taxon hypotheses, species hypotheses, metabarcoding

## Abstract

Here, we describe the taxon hypothesis (TH) paradigm, which covers the construction, identification, and communication of taxa as datasets. Defining taxa as datasets of individuals and their traits will make taxon identification and most importantly communication of taxa precise and reproducible. This will allow datasets with standardized and atomized traits to be used digitally in identification pipelines and communicated through persistent identifiers. Such datasets are particularly useful in the context of formally undescribed or even physically undiscovered species if data such as sequences from samples of environmental DNA (eDNA) are available. Implementing the TH paradigm will to some extent remove the impediment to hastily discover and formally describe all extant species in that the TH paradigm allows discovery and communication of new species and other taxa also in the absence of formal descriptions. The TH datasets can be connected to a taxonomic backbone providing access to the vast information associated with the tree of life. In parallel to the description of the TH paradigm, we demonstrate how it is implemented in the UNITE digital taxon communication system. UNITE TH datasets include rich data on individuals and their rDNA ITS sequences. These datasets are equipped with digital object identifiers (DOI) that serve to fix their identity in our communication. All datasets are also connected to a GBIF taxonomic backbone. Researchers processing their eDNA samples using UNITE datasets will, thus, be able to publish their findings as taxon occurrences in the GBIF data portal. UNITE species hypothesis (species level THs) datasets are increasingly utilized in taxon identification pipelines and even formally undescribed species can be identified and communicated by using UNITE. The TH paradigm seeks to achieve unambiguous, unique, and traceable communication of taxa and their properties at any level of the tree of life. It offers a rapid way to discover and communicate undescribed species in identification pipelines and data portals before they are lost to the sixth mass extinction.

## 1. Introduction

Systematics face the daunting task of classifying our living world into monophyletic groups in a hierarchical Linnean system. This work is more complete for some groups of organisms than for others. For instance, estimates suggest that more than 70% of all extant plant species but only 4% of the upwards of 3.8 M estimated number of fungal species have been described [1,2]. The study of fungi—mycology—is further compounded by the nature of fungal life, which we now know to be largely subterranean, substrate-dwelling, or otherwise inconspicuous, typically with only sporadic physical manifestations of the underlying species. Morphology constituted the primary information source for fungal systematics up until the introduction of molecular tools in mycology in the early 1990s. Already from the onset the molecular data hinted at the complexity of the evolutionary history of fungi. Convergent evolution as well as multiple gains and losses of characters and character states were found to permeate the fungal tree of life, and hitherto unrecognized species and higher taxa seemed to abound. As ever-increasing swathes of the fungal kingdom were subjected to molecular scrutiny, it became painstakingly clear that very substantial modifications to the classification and naming of fungi and fungal groups were looming on the horizon [3].

Today, molecular data form an indispensable cornerstone of mycological systematics. That is not to say that all DNA-based studies in systematics produce identical results and propose comparable classifications, however. Taxon sampling, choice of genetic markers, and differences in analytical approaches combine to give rise to diverse and partially incongruent competing classifications (e.g., [4,5]). Which, if any, of the many proposed classifications is correct may simply be too early to say. It is, therefore, not surprising that no unified, joint classification underpins the many online resources that house and curate mycological data. Indeed, a user is likely to find many differences when comparing the classifications used in, e.g., GenBank [6], MycoBank [7], UNITE [8], CoL/GBIF [9], and BOLD [10]. The classifications of each of these many resources evolve more or less independently over time; some resources seek to offer the latest developments and thus incorporate the results of all recent studies in systematics, whereas others prefer to adopt only the most well-vetted aspects of the new classifications. This has led to a plethora of classification systems that is hard to overview and where interpretation what a given taxon (name) comprises in the different systems is impossible. The meaning of the statement “following the classification of resource X” will, potentially, mean different things depending on whether it is written in January, in June, or in December, and there would be no immediate way of finding out what the author had in mind in the first place.

During the development of the UNITE database for molecular identification of fungi we recognized the need to be able to lock all classifications accepted at any time digitally, and to provide a timestamped unique persistent identifier (PID)—such as a digital object identifier (DOI)—for them. The classification system would then be made available online for others to use—but also for others to modify and augment. The modified version would then be made available online, alongside the original one, under the same conditions. Such an approach would solve both the back-tracking problem and the issue with precise identification of classification systems. If this resource was provided in a software environment that was relatively standardized and easy to work with, then this dynamic approach to classification systems could serve as an arena to experiment with different classifications and their consequences for fungal systematics and nomenclature. The system would at all times be both communicable through a PID and open to further modification, the latter in either a public or in a more restricted way. Maintaining parallel circumscriptions of a species, or several competing classifications of, e.g., *Fusarium* or Basidiomycota, would no longer be a problem. Such a communal resource management system could conceivably facilitate the ulterior chiseling out of a grand and widely agreed-upon classification of the kingdom of fungi at all taxonomic levels. The first step toward the taxon datasets communicated via PIDs was launched by UNITE in 2011 when the first versions of the SH datasets were published online [11].

In this paper, we expand the SH concept and introduce the taxon hypothesis (TH) paradigm as an open and reproducible approach to the construction, identification, and communication of taxa as datasets. We show how the TH concept is implemented in a molecular context in the UNITE database as datasets of individuals (specimens and other biological samples) and their characters (sequences and other traits), and we explain why we think the TH approach may be conducive to the synthesis of a classification system for all fungi. The TH concept is nevertheless applicable across systematics and the tree of life. We showcase a range of examples from other eukaryotic groups in the context of public nuclear ribosomal internal transcribed spacer (ITS) sequences.

## 2. Materials and Methods

### 2.1. Founding Elements of the Taxon Hypothesis Paradigm

The three major elements influencing the formation of the taxon hypothesis (TH) (Appendix A) paradigm can be seen as building blocks of the overall idea:

1. The theory of scientific hypotheses and their falsification has greatly influenced the development of the TH paradigm (e.g., [12,13]). However, we acknowledge that the best taxonomic papers published before Popper’s works included taxonomic descriptions amenable to falsification in the sense that high-quality taxon descriptions contain list of studied specimens and their properties. Therefore, other researchers can restudy specimens lodged in public collections and falsify primary taxon descriptions. However, our understanding that taxon descriptions can be viewed as proper scientific hypotheses emerged only when the theory of falsification (and the debate that ensued) became available. The TH paradigm seeks to capture scientific hypotheses and to provide a venue for their falsification through all levels of the tree of life and across time. The aim of this paper is not to discuss the limitations of falsification—we just want to emphasize its influence on the TH paradigm in its early stages of conception in the late 1980s.
2. Zavadski’s book [14] features rich information on species criteria (SC), namely, morphological, biochemical, geographical, ecological, genetical, and physiological properties of the species. He had a view that all—or at least most—of these criteria must be used for the discrimination of species. Zavadski also introduced the practical species standard (PSS), which is a set of instructions on how species boundaries and content are defined. His view was that species theory or species concepts must be kept separately from PSS. Our TH paradigm takes into consideration that (1) all species criteria should be considered when delimiting species; and (2) the paradigm can be accompanied with one to many practical species standards. The UNITE identification and communication system is one example of the PSS of the TH paradigm. Therefore, this discussion of the TH paradigm is illustrated by examples from the UNITE system. These examples can also be called UNITE PSS sensu Zavadski. The major difference is that the TH paradigm is widened to include taxa at all levels, not only species.
3. Dallwitz [15,16] developed the Descriptive Language for Taxonomy (DELTA), which is a computer-based system for encoding and management of taxonomic characters or attributes. The DELTA format allows the user to atomize all properties (characters sensu Dallwitz) of the biological individuals and taxa, and then build datasets of encoded properties called Items. Originally, the Items were conceived for the computational processing of taxon descriptions and for the automated generation of identification keys. THs have some similarities to the DELTA system. They, too, are datasets of taxon properties similar to DELTA Items. The major difference is that the TH datasets include individuals, and that properties are always attached to the individuals. The DELTA Items, however, are taxon descriptions, where the properties of the individuals are summarized such that the property of each individual is lost. An exception would be when Items include properties of a single individual. In the TH paradigm, individuals and their properties are conceptually free to float between datasets. They may appear in another TH when new hypotheses are computed. However, the same individuals in different THs are linked through the unique PIDs. This feature is also implemented in the UNITE system.

There are many other elements which influenced the development of the TH paradigm. Advances in information technology and molecular biology are the most prominent of these. Databases and open data of the International Nucleotide Sequence Database Collaboration (http://www.insdc.org) played an important role in the development of the TH paradigm and particularly for the development of the UNITE system.

### 2.2. UNITE—A Practical Example of the Taxon Hypothesis Paradigm

#### 2.2.1. UNITE SH Calculations

For the calculations of SHs, two datasets of eukaryote rDNA ITS sequences were used: an International Nucleotide Sequences Database Consortium (INSDC) dataset [17] downloaded via the NCBI data portal [18], and the UNITE database [19] with ITS sequences not yet available through the INSDC. The datasets were merged, and six computing steps were executed: (1) quality filtering; (2) ITS extraction—for the remaining sequences, all genes (SSU, 5.8S, and LSU) and spacer regions (ITS1, ITS2) were separated using ITSx v. 1.1.2 [20] using a two-step extraction (first Fungi only, then all other taxa); (3) additional quality filtering using UCHIME and VSEARCH to filter out chimeric sequences, and custom Python scripts to exclude sequences containing more than six ambiguous nucleotides (sensu [21]) from further analysis; (4) USEARCH clustering (clustering step 1)—full ITS region for sequences passing the cleaning steps were submitted to USEARCH v10.0.240 [22] analysis for hierarchical clustering at a 80% similarity threshold. The first clustering step produced compound clusters and singletons. Compound clusters include closely related species, which usually do not comprise a full genus; (5) blastclust clustering (clustering step 2), where single clusters from clustering step 1 were clustered further using blastclust version 2.2.26 [23] on different distance threshold levels (0.5–3.0% corresponding to 97–99.5 percent identity). The outcome of this step are datasets of SHs; (6) aligning sequences of SHs from step 5. The multiple sequence alignment program MAFFT v7.215 (default settings) [24] was used to align the sequences.

#### 2.2.2. UNITE TH Construction

Construction of the UNITE TH datasets is based on SHs, which are connected to a predefined hierarchical taxonomic system called the taxonomic backbone. Ideally, the taxonomic backbone offers resolution all the way down to the species level, but in practice this will vary across the fungal tree of life (see Results 3.4.1.–3.). Genus-level THs were constructed from SHs that carry the same genus name. Order-level THs were constructed from all genus-level THs that according to the taxonomic backbone belong to this order. The same technique was used at higher levels. The TH datasets contain individuals and their properties; in addition, individuals link to lower and higher level THs where they belong. For communication purposes, every dataset of the TH release received a unique DOI. If SHs were only identified at genus or higher level, the TH construction started from the level where the SH was identified (see Results 3.4.1.). Each TH is a dataset that contains all individuals and their ITS sequences from lower level THs and SHs. In addition, each dataset includes a distribution map, ecological traits, and links to other associated THs. The DOI page of a TH also provides all associated ITS sequences in the FASTA format [25] for download (see Results 3.4.3.).

### 2.3. PlutoF—The Data Management Platform for Taxon Hypotheses

UNITE datasets are hosted by the biological data management platform PlutoF, which implements most of the features of the TH paradigm ([26], https://plutof.ut.ee/). In PlutoF, users can handle very different data types such as classifications, collection (including living collection) specimens, other environmental samples (e.g., water, soil, and wood), locality data, DNA sequences, laboratory experiments, and literature as a single dataset. It is an open data management platform that provides online services for scientists, labs, institutions, research collections, citizen scientists, and others. The focus is on biodiversity data covering taxonomy, ecology, monitoring, conservation, and genetics. Users can manage their biodiversity datasets through a full data life cycle—from uploading to publishing and archiving in the FAIR format [27]. PlutoF follows major data standards developed in this field, notably Darwin Core [28] and Minimum Information about any (×) Sequence (MIxS; [29]). Therefore, data are easy to export to other repositories and data portals such as GBIF and the INSDC. Since PlutoF is a part of the DataCite consortium [30], it is also possible to ask for DOIs for research datasets managed in the system.

PlutoF also allows cross-border collaboration and development of common databases, which is a feature often used by research networks such as UNITE. Users can use their ORCID or other social media accounts to access the infrastructure.

### 2.4. Implementation of the UNITE Species Hypothesis System in GBIF

The GBIF backbone taxonomy [31] is a single, synthetic management classification with the goal of covering all names that GBIF handles. This backbone underpins GBIF and allows it to normalize nomenclature of diverse species occurrence data with all their name variants. Traditionally, this backbone has been based solely on Latin scientific names. However, in recent years, a gap has opened between the set of species formally described and the set of provisional species identified and understood by clustering individuals into operational taxonomic units (OTUs; [32]) based on DNA barcoding. The UNITE SH concept provides a solid communication system for described as well as as-yet undescribed fungal species and sheds light on scientific name usages of different reproducible taxon concepts. This system offers scientific resolution also in the cases where Latin names do not. For instance, there are countless examples of where a Latin scientific species name is applied to several different species (hypotheses), or where one species (hypothesis) has been labeled with several different Latin scientific names. UNITE’s species hypotheses was the first set of non-Latin names integrated in the GBIF Backbone Taxonomy in 2018 [33]. This inclusion allowed indexing of occurrence and sampling event data, taxonomically annotated with SH identifiers and published through GBIF. Technically, this is implemented based on a taxonomic checklist [34] published by UNITE to GBIF, and included in the GBIF backbone build. SHs are presented as unranked children of their nearest Latin name parent, which determines the classification of SHs within the GBIF Backbone Taxonomy. This largely corresponds to the connection of SHs to the UNITE taxonomic backbone (see Results 3.3.)

Following the same approach, the GBIF backbone taxonomy was further amended with barcode index numbers (BINs; [35]) from the International Barcode of Life database in 2019 [36]. A checklist dataset [37] was published based on the taxonomic annotation of all individuals within each public BIN. Taxon names for BINs were assigned following the main principles of the SH taxon name assignment (see Results 3.2.2.), but an 80% consensus rule was applied to keep e.g., a species level name assignment if four out of five names in the BIN were equal, rather than assigning the BIN to a higher taxon. Today, the GBIF taxonomic backbone includes 158,946 SHs and 466,069 BINs. GBIF works with both UNITE and iBOL on faster automated updates for a more agile reflection of OTUs in GBIF, and, consequently, better indexing of DNA-derived data.

## 3. Results

In the following paragraphs, we describe the TH paradigm and in parallel give examples of how it is implemented in the UNITE system.

### 3.1. Construction of Species Hypotheses

**Figure 1:**
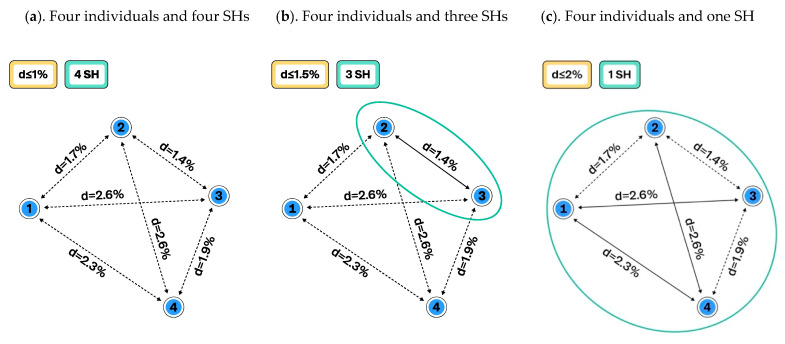
Schema showing how changing the distance threshold between species hypotheses (SHs) influences our comprehension of species delimitation and intraspecific variation: (**a**). Distance threshold between species is set to ≤ 1%. All four individuals belong to different SHs, and the intraspecific variation is zero; (**b**). The distance threshold is set to ≤ 1.5% and three SHs are delimited. Because the distance between individuals 2 and 3 is less than 1.5%, they fall into the same SH, which has an intraspecific variation of 1.4%. Two other individuals remain distinguished as separate species; (**c**). The distance between SHs is set to ≤ 2% and now all four individuals belong to a single SH with an intraspecific variation of 2.6%. Some distances are greater than 2%, but these individuals have sister individuals with a distance of less than 2%.

**Figure 2:**
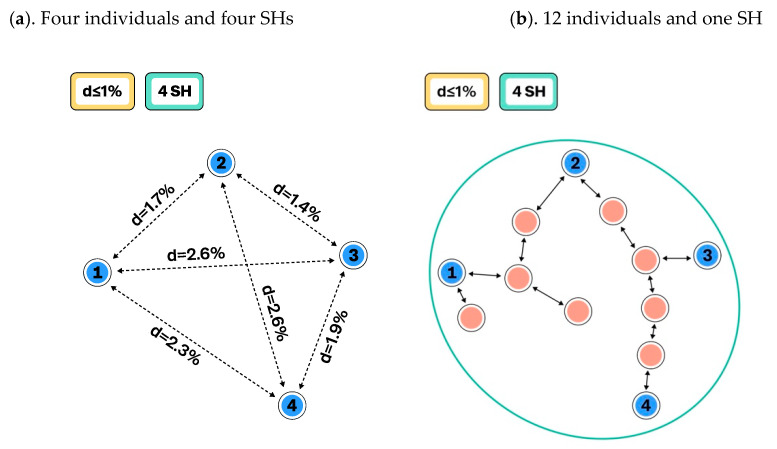
Schema showing how the number of studied individuals influences the composition of species hypotheses (SHs) and intraspecific variation: (**a**). The distance between SHs is set to ≤1%, and all four individuals belong to different SHs. The intraspecific variation is zero (from Figure 1a); (**b**). Eight closely related additional individuals were studied (red dots) and they changed the composition and intraspecific variation of the SHs. Now every individual has a sister individual with a distance of less than 1%. Therefore, individuals which formed four different SHs (**a**) are merged into a single SH when the same distance between SHs, namely, ≤ 1%, is used. However, infraspecific variation is as high as 2.6%.

**Figure 3:**
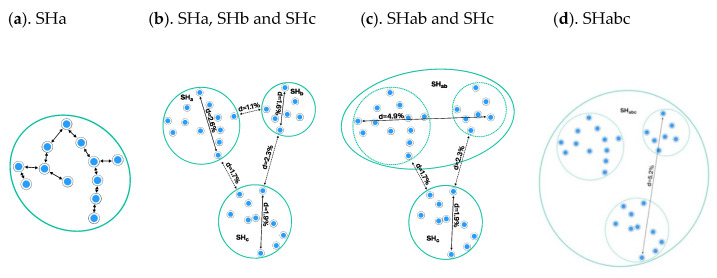
Example with higher numbers of studied individuals and species hypotheses (SHs). (**a**). The distance between SHs is set to ≤1%, and all 12 individuals belong to a single SH (SHa) with an intraspecific variation of 2.6% (from Figure 2b); (**b**). Twenty more individuals are studied, which forms two separate SHs (SHb and SHc) when the distance between SHs is set to ≤1%; (**c**). When the distance between SHs is set to ≤1.5%, SHa and SHb merge because the smallest distance between individuals in SHa and SHb is 1.1%. Intraspecific variation of SHab is 4.9% and SHc is 1.9%; (**d**). When the distance between SHs is set to ≤2%, SHc merges with SHab because the distance between the two closest individuals of SHa and SHc is 1.7%. Intraspecific variation of the SHabc is as high as 5.2%.

The species hypotheses represent the most resolved (lowest) level of the taxon hypotheses. The construction of the SHs can be based on any single criterion or combination of them. For example, SHs can be inferred from morphological, anatomical, or genetical criteria or by combining them into a single dataset. The important prerequisite is that all traits or characters (from now on referred to as traits) are atomized to make it possible to calculate distances between individuals in automated way. Individuals, their traits, and all past changes must be databased in order to communicate SHs as well as higher-level THs with PIDs in research papers and data portals as machine readable data. The current TH paradigm uses the SH delimitation distance thresholds (Figure 1, Figure 2 and Figure 3). However, intraspecific variation as an SH threshold can also be utilized, but this method will be explored elsewhere.

Figure 1 demonstrates how changing the distance threshold between SHs can change the number of observed species and their intraspecific variation. The number of studied individuals may have a similar effect (Figure 2), but this is not an issue if all individuals and their traits are carefully atomized and databased. In the latter case, all changes in SHs can be traced, compared, and communicated in an automated way. Such a system allows the use of different distance threshold values while maintaining comparability of results across studies. Figure 3 demonstrates how the distance threshold values ≤ 1.0%, ≤ 1.5%, and ≤ 2.0% influence the number of SHs and intraspecific variation based on the same dataset of individuals. Biodiversity studies where taxon recoveries are communicated through different distance thresholds are still comparable when the same datasets of SHs are utilized for the identification.

#### UNITE Example: Computation and Visualization of SHs in UNITE

For the inference of SHs, UNITE relies on rDNA ITS sequences (see Material and Methods and [8,11,26,38]). Today, a strong case can be made for DNA sequences as the best traits for the inference of SHs and THs; DNA sequences are atomized from the beginning (four nucleotides) and are easy to database and analyze computationally. Solutions for using morphology and other traits for the computing SHs are in progress. UNITE version 8.2 comprises 251,772 eukaryote SHs at the ≤1.5% distance threshold value; of these, 102,100 (41%) belong to fungi.

**Figure 4:**
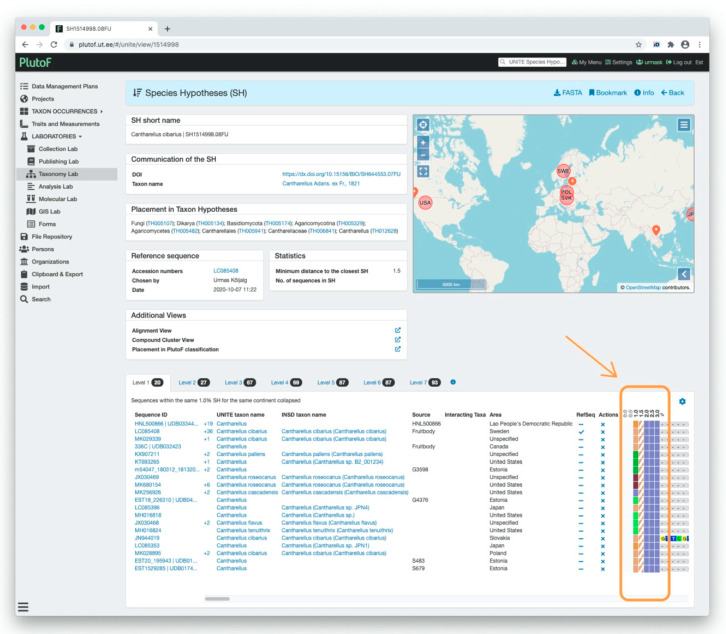
Visualization of the *Cantharellus cibarius* species hypothesis (SH) in UNITE 8.2. The color patterns in spreadsheet format (orange rectangle) show that all individuals belong to the same SH when the distance between SHs is set to ≤1.5% (orange arrow). When the distance is set to ≤1.0%, the same individuals are spit in six SHs.

Figure 4 presents an overview of the individuals and alignment of their ITS sequences of the SH dataset in PlutoF (Figure 4). It also includes panels for the SH taxon name and DOI, links to the higher level THs, reference sequence, statistics, and additional viewing options. In the figure, the ≤1.5% distance threshold value produces an SH of 93 individuals (=DNA sequences) with the species name of *Cantharellus cibarius* Fr. At the ≤1.0% distance level, this SH is split into six SHs which have different species names available.

### 3.2. Communication of SHs via Persistent Identifiers (PID) and Taxon Names

**Figure 5:**
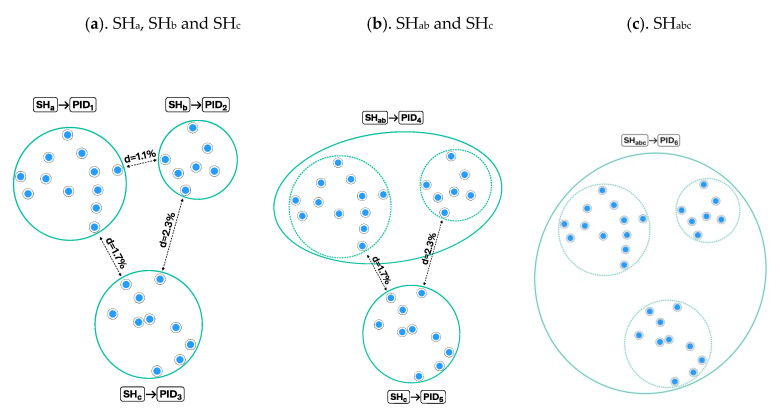
Assigning persistent identifiers (PIDs) to the species hypotheses (SH) from Figure 3b–d. (**a**). The distance is set to ≤1.0%, and three SHs (SHa, SHb, and SHc) are assigned unique PIDs (PID1–3); (**b**). The distance between SHs is set to ≤1.5%, and two new PIDs are assigned to SHab and SHc. The content of PID5 and PID3 is identical but the distance is not; hence, the new PID assignment is necessary; (**c**). The distance between SHs is set to ≤2%, and as a consequence SHc merges with SHab. Only one PID6 is assigned—namely to SHabc.

It is important that every SH dataset can be communicated via a unique PID. If the content of the SH dataset changes due to the addition or removal of individuals and traits, then a new PID must be assigned. Figure 5 demonstrates that the same individuals may form as many as six different SHs, each of which has a unique PID, since the distance between species is set at three different levels.

SHs are datasets of individuals which in the case of specimens can be studied and identified by experts. Such identifications of individuals are used when choosing a taxon name of the SH. If an SH dataset includes the type specimen, then that name is used for the communication of the SH. When data on several type specimens are in the same SH dataset, then the species name is chosen according to the rules set by the relevant nomenclatural code.

#### 3.2.1. UNITE Example: Communication of SHs via Digital Object Identifiers (DOIs)

**Figure 6:**
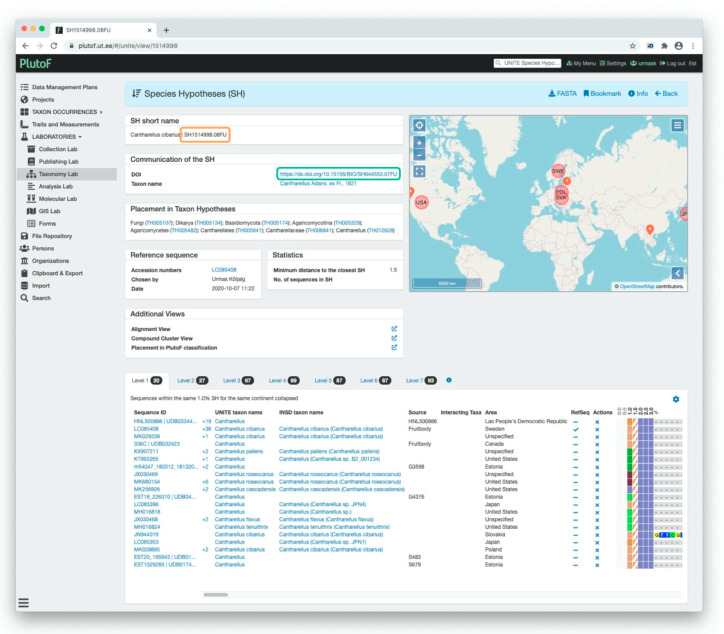
Communication of the species hypothesis (SH) in UNITE 8.2. Digital Object Identifier (DOI) is used as a unique PID for the publishing and communicating of SH datasets. On the SH page, the full DOI address of the dataset is shown (green rectangle) as well the abbreviation (orange rectangle).

The UNITE system uses DOIs as a PIDs to communicate SH datasets. The DOI link in Figure 6 takes the user to the official web page of this SH dataset. Since 2015, UNITE has released more than half a million DOIs of SH datasets. Individuals in the datasets also have PIDs, which allows the user to track their location in different UNITE versions. However, by default, UNITE uses external PIDs assigned to individuals (sequences and specimens) by INSDC, natural history collections, or biobanks. If individuals have no unique external PID, then UNITE provides its own identifiers.

#### 3.2.2. UNITE Example: Communication of SHs via the Taxon Name

**Figure 7:**
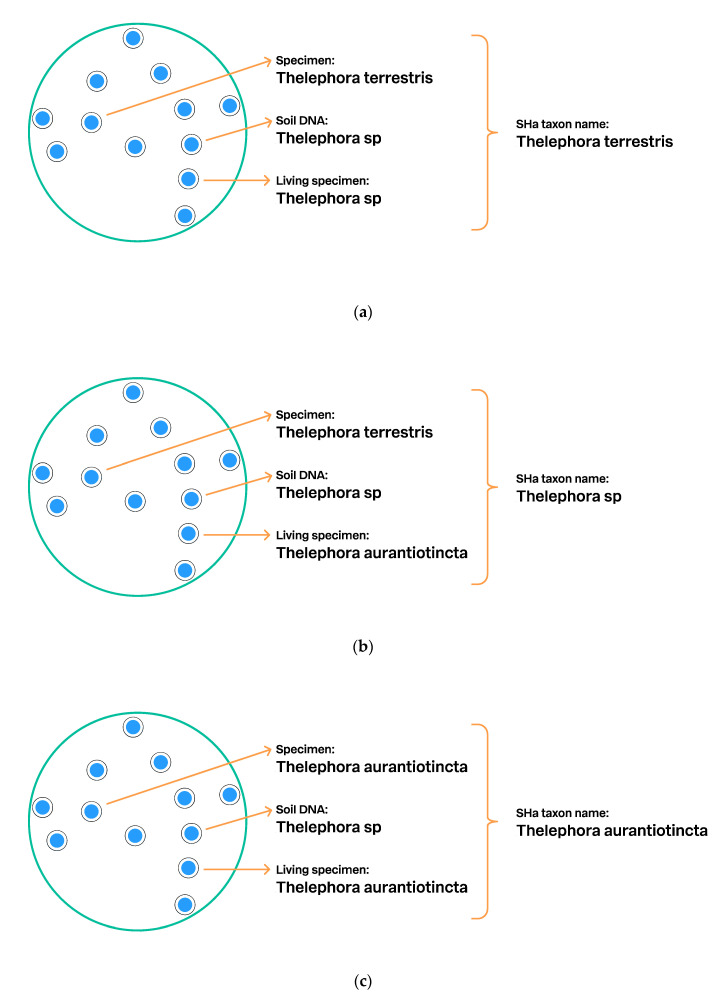
Schema showing how the UNITE script assigns a taxon name to SHa: (**a**). One specimen was identified as *Thelephora terrestris*, a living specimen as *Thelephora* sp., and DNA sequence from soil as *Thelephora* sp. The UNITE algorithm chooses the full species name as the SHa name in the absence of direct conflict; (**b**). The same individuals, but here a new identification of the living specimen, namely, *Thelephora aurantiotincta*, was added to the database by a power user. Two different species names will cause a nomenclatural conflict, and the script chooses the genus name *Thelephora* as a consensus name for SHa. The content (individuals and traits) of the SHa remains identical, as does the digital object identifier (DOI). The same DOI allows stable communication of the SHa and its individuals even if the taxon name changes; (**c**). Again the same individuals, but the specimen in the collection was restudied by an expert, who provided a new identification: *Thelephora aurantiotincta*. Now the UNITE script will choose *T. aurantiotincta* as the name for SHa.

Individuals, such as specimens in the UNITE SHs datasets, can be studied by experts and identified at a species or higher level. These identifications are used when deciding upon a taxon name for the UNITE SH. If the SH dataset includes the type specimen, then that name will be used as the name of the SH. When data on several type specimens are in the same SH dataset, then the species name is chosen according to the relevant nomenclatural code. The UNITE system gives power users the ability to make such annotations (decisions) through the PlutoF platform. Oftentimes, individuals in the same UNITE SH are assigned with alternative species or higher-level taxon names. Figure 7 shows a case where the specific software script makes nomenclatural decisions for the SHs.

### 3.3. Connecting SHs to the Taxonomic Backbone

**Figure 8:**
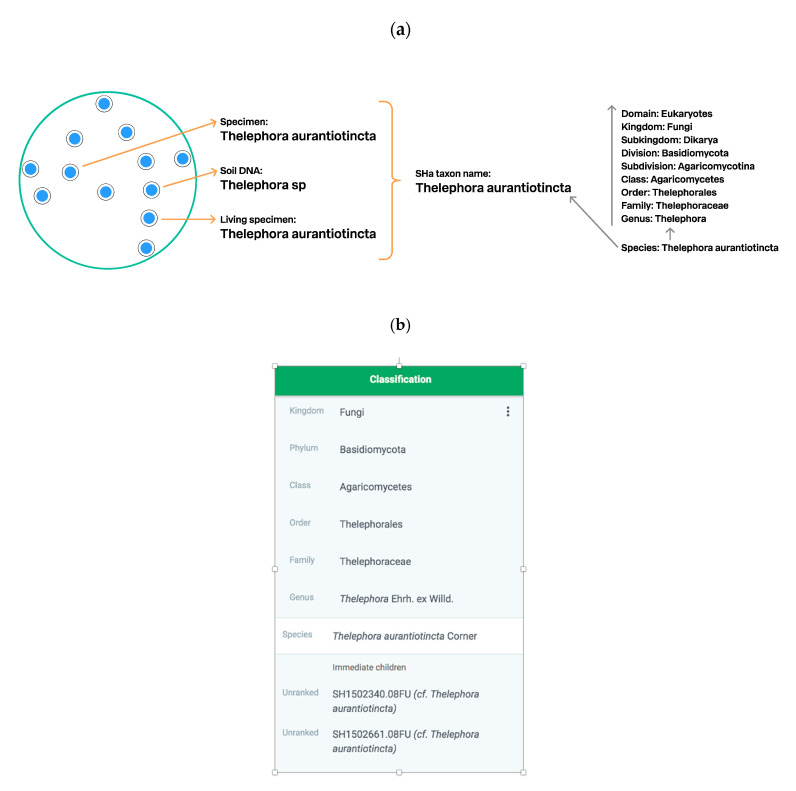
Schema of bridging of SHs and the taxonomic backbone. (**a**, **left**). *Thelephora aurantiotincta* is chosen as a taxon name of the SHa (from Figure 7c); (**a**, **right**). Taxonomic backbone showing the lineage of the *T. aurantiotincta*. The blue line demonstrates the connection between SHa and the taxonomic backbone. The black line shows that SHa is connected to all levels of the taxonomic backbone. (**b**). Classification of *Thelephora aurantiotincta* in the GBIF taxonomic backbone with two UNITE SHs. Both SHs can be published in GBIF as separate taxa.

In the previous paragraph, we described how the name of the SH is chosen and showed one example of how the UNITE script chooses taxon names automatically (Figure 7). The species or higher-level taxon name can be used to connect SHs to the taxonomic backbone if it features this name and any associated higher hierarchy levels. Figure 8 shows the connections between SHs, species names, and the taxonomic backbone. In addition, we show an authentic example of the same species in the GBIF classification (Figure 8). There are two UNITE SHs under the species name *Thelephora aurantiotincta* Corner, and the system allows both SHs to be published in the GBIF data portal despite the fact that they carry the same species name. One of the most striking examples in UNITE is the well-known human pathogen species *Aspergillus fumigatus* Fresen. (Table 1). It has 183 SHs but only one SH includes a type of the species (SH2189906.08FU). However, the TH paradigm allows the remaining 182 UNITE SHs to be published and communicated in GBIF data portal and elsewhere as a unique species. This would not be possible via the species name of *A. fumigatus*.

### 3.4. Construction of Taxon Hypotheses at Higher Levels

#### 3.4.1. Using a Taxonomic Backbone

**Figure 9:**
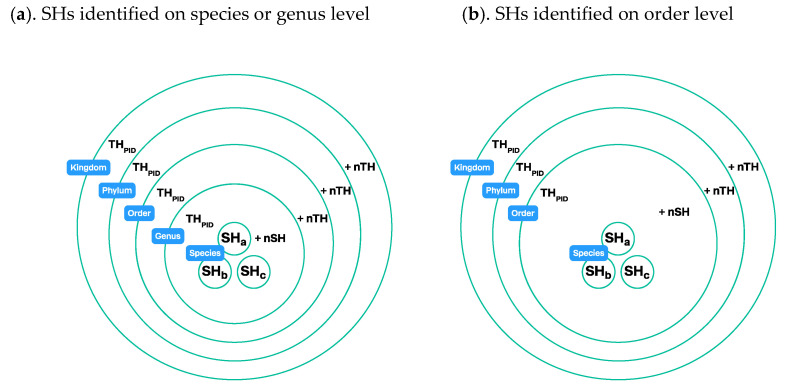
Construction of taxon hypotheses connected to the taxonomic backbone via taxon names: (**a**). Names of the species hypotheses are connected to the taxonomic backbone (see Figure 8). Every TH is pieced together from lower-level datasets of THs. A genus-level TH is constructed from SHs that carry this particular genus name. An order -level TH is constructed from all genus-level THs that according to the classification belong to this order. The same technique is used at higher levels. Every TH has a unique persistent identifier (PID). More classification levels can be added to the communication system if needed; (**b**). SHs are identified at the order level. The species- and genus-level placement in the classification are unknown, and the SHs are connected to the order-level TH.

There are two approaches to constructing taxon hypotheses. In the first approach, THs are constructed by pooling all lower level THs (individuals and their properties) that belong to this taxon. In this method, a taxonomic backbone is necessary because taxon names determine which THs should be pieced together at the next level (Figure 9). UNITE THs are constructed using this method. At first, SHs are computed based on rDNA ITS sequences. SHs are then connected to the taxonomic backbone based on taxonomic identities assigned by experts, and THs are thus formed according to the selected classification. In principle, different classifications can be used at the same time, but currently, UNITE is calculating THs only for one classification. We emphasize that the THs, including the SHs, are always datasets of individuals with their atomized traits.

#### 3.4.2. Using Operational Taxonomic Units

**Figure 10:**
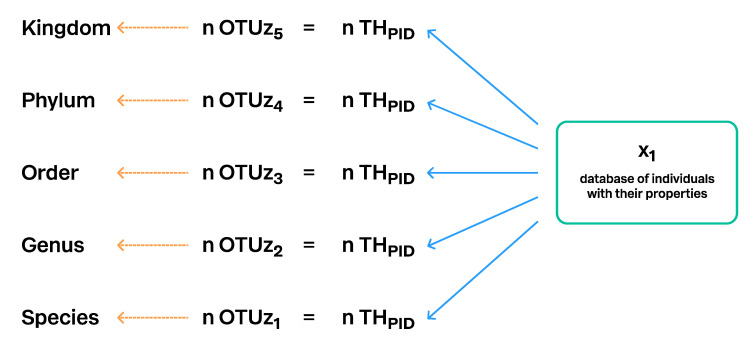
Construction of Taxon Hypotheses computed directly from the database of individuals and their properties. Different OTU levels are computed by changing the distance or similarity threshold and by using properties which are more conserved or more variable. Every TH has a unique persistent identifier (PID). The number of the OTUs (n) of the specific level is always equal to the number of THs of the same level. OTUz_5_ is the Operational Taxonomic Unit of the hierarchical level 5; z shows that OTUs of the different levels are computed from the same database of individuals. However, the properties of the individuals may vary.

In the second approach, THs are computed independently for different levels based on the same dataset (individuals and their properties). For this method, neither taxon names nor a taxonomic backbone is necessary. Only properties (traits) of the individuals are needed for the computation, and THs are communicated via PIDs only. For such THs whose level in the classification is unknown, the term operational taxonomic unit (OTU) has been coined in microbiology [32,39] and widely used in research papers since then. This approach can also be combined with a taxonomic backbone; ideally, the OTU—consisting of one to many THs—is connected to the specific classification level after the computation of THs. Effectively, then, THs are no longer OTUs because their position in classification is defined. THs can then be communicated via PIDs as well as via taxon names at some given level of resolution (Figure 10).

#### 3.4.3. UNITE Example: Taxon Hypotheses

**Figure 11:**
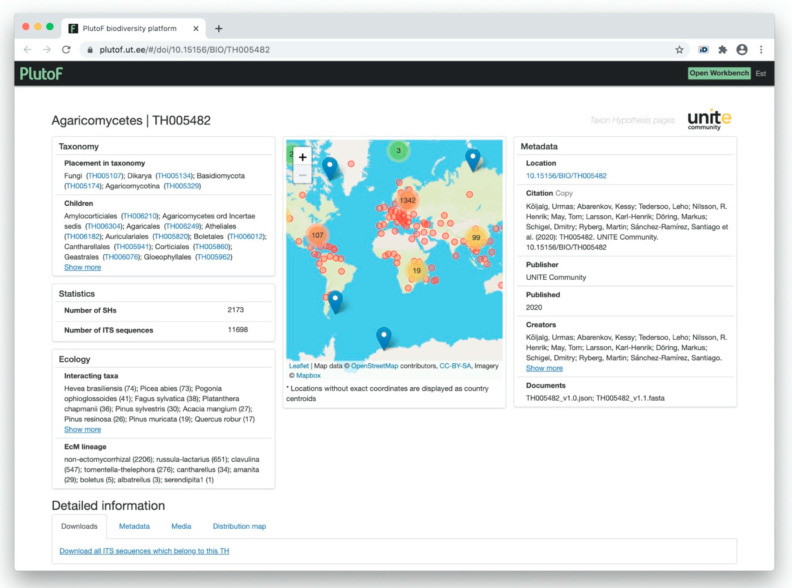
Visualization of the taxon hypothesis of the fungal class Agaricomycetes (TH) in UNITE 8.2.

Table 1 provides several examples of UNITE THs including SHs. Three examples are for Fungi and two examples represent Amoebozoa and Viridiplantae. The THs are assigned with unique DOIs, and the corresponding link will open the respective web page of the dataset (Figure 11). The short code of the TH consists of the acronym TH and a six-digit number. The DOI pages provide options to download all ITS sequences of all individuals in the current TH as a FASTA file. Additional services will be available in future UNITE versions.

### 3.5. Communication of Taxon Hypotheses

**Figure 12:**
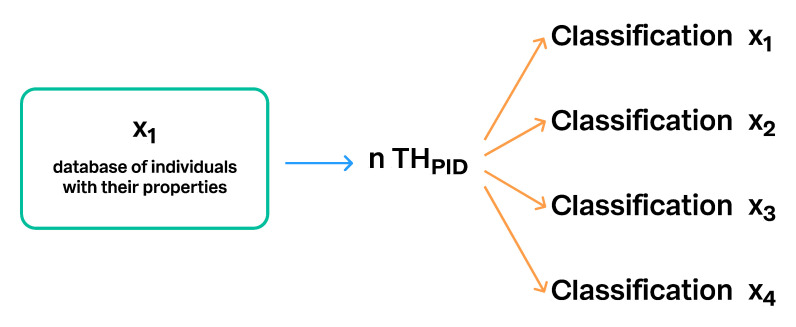
The same Taxon Hypothesis with a unique PID can be connected to an unlimited number of different classifications. This is one way to connect different taxonomic backbones and making them comparable. Classification x_1_ to x_4_ designate different classifications.

**Figure 13:**
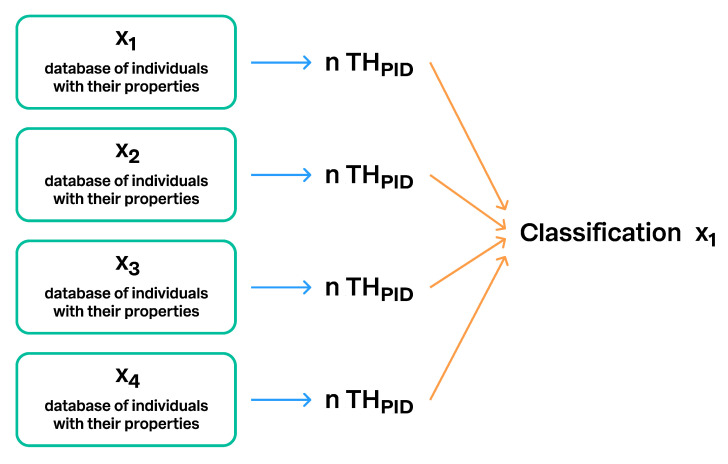
Single classification can be connected at the same time to THs, which are based on different databases of individuals and their properties. The same taxonomic backbone can populate many different THs connected to the same taxon name, making them in this way comparable.

**Figure 14:**
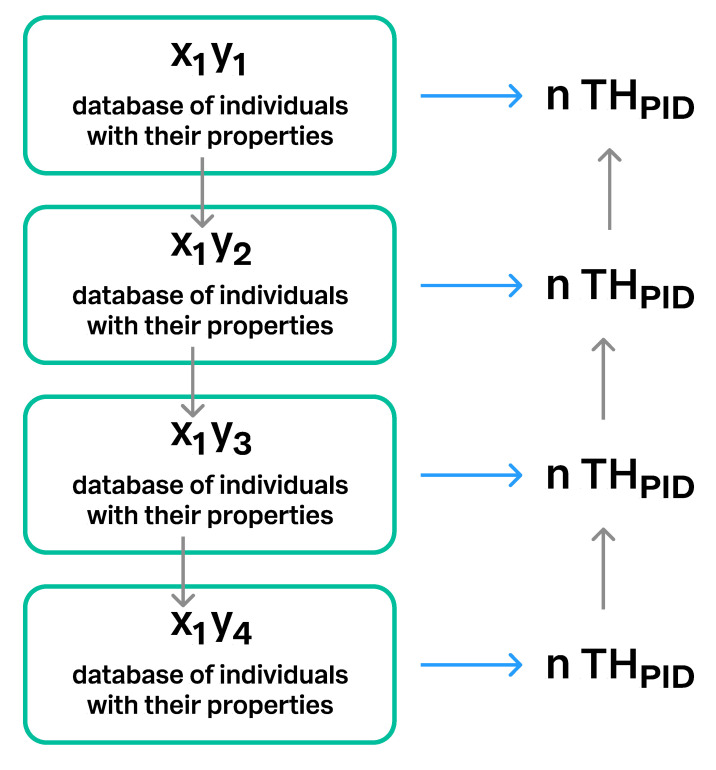
Connections between different versions of the same database. The same database may have different versions when new individuals and/or properties are added to the data. x_1_ = the database, y_1_–y_4_ = four different versions of the database x_1_.

The TH paradigm was designed to be flexible. Every individual (specimen/DNA sequence) may belong to an indefinite number of different THs, and every TH may belong to an indefinite number of different classifications. However, the rule is that individuals and their datasets (=THs) must be digitally traceable and comparable through the space of biological data in any fixed time. The schemas in Figure 12, Figure 13 and Figure 14 demonstrate multiple connections between individuals, THs, and taxonomic backbones.

### 3.6. Discovery of Formally Undescribed Species

**Figure 15:**
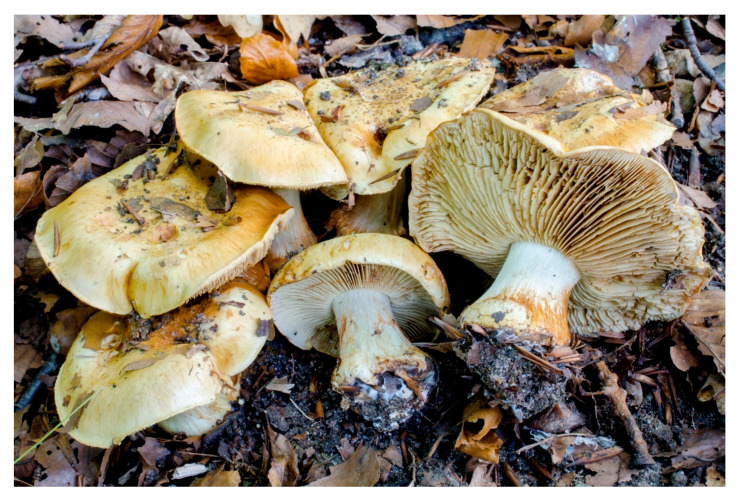
In situ photo of *Cortinarius koldingensis*. Marielund, Kolding, Denmark. 13 October 2013. (photo Tobias Guldberg Frøslev).

One crucial feature of the TH paradigm is that it has the power to discover formally undescribed species and provide communication tools for them. See also the discussion on this matter in *Science* [40,41]). Table 2 provides select examples of such cases where the UNITE SH system recognized species which were formally described only later. For example, the fungal species *Cortinarius koldingensis* was published in 2015, but it was communicable as a UNITE SH already in 2013 (Figure 15; [42]).

Based on indirect analyses, the current version of UNITE 8.2 includes thousands of SHs, which represent undescribed species [45]. This renders the UNITE system something of a gold mine for the discovery of new species, particularly as data evincing new species keep coming in through eDNA studies.

## 4. Discussion

### 4.1. Liaison of the TH Paradigm with Taxonomy and Nomenclature

The most important cornerstone of the TH paradigm is that taxa are treated as a dataset of individuals and their properties. As we already noted, high-quality taxonomic research handles species in the same way. Over the last several centuries, standardized group-specific sets of characters (traits) became established for the description of novel species. Since around the beginning of the 20th century, published descriptions of species began to include comprehensive lists of specimens examined. Furthermore, across the examined specimens, quantitative characters are routinely described on the basis of ranges and other metrics and qualitative characters are expressed as the sets of states of particular characters and their prevalence. The reproducibility of species discrimination was assured because specimens were lodged in public reference collections. Therefore, scientists could restudy these specimens and verify or falsify previous research. Depending on the taxonomic group, a specimen represents either a single individual (for larger animals and many plants) or is a collection of individuals from one location that may or may not be genetically identical (in the case of mushroom sporophores).

In the 1970s, digital taxon description methodologies emerged (e.g., DELTA by [15]) which improved greatly the possibility to create large datasets that accurately described trait variation within and between taxa. The current high-quality taxon trait datasets are machine readable and follow major international biological data standards such as DarwinCore [46], ABCD [47], MIxS [48], and many other standards developed by TDWG [49]. In addition, some online journals are introducing innovative features in publishing taxon descriptions such as live links to specimen metadata and machine-readable taxon descriptions marked up in xml which are readily exportable to databases and verified (e.g., in journals published by Pensoft such as MycoKeys, etc.; [50]).

Given the widespread citation of included specimens and the presentation of comprehensive trait descriptions, we can conclude that most elements of the TH paradigm are already in place except one. This is the way how we communicate taxa, in particular taxon circumscriptions. International codes of nomenclature set rules on how the names of taxa are communicated. Broadly, the communication system of the codes is built on species being anchored to preserved specimens called types. Types are not necessarily “typical” for the species, but are permanently available for confirming traits described originally or investigating novel traits, especially where there is dispute about the application of a name. Codes take care that the species name (=identifier) is globally unique. Additionally, some codes mandate or encourage digital registration of taxon names, and therefore, names carry in addition a unique persistent identifier (PID). Such registration of taxon names is for example mandatory in mycology [51,52]). The major drawback of such a communication system is that the name is tied to a single specimen or collection. However, species circumscriptions are ideally based on multiple individuals and are dynamic in time. Indeed, identification of further individuals of a taxon can only widen the range of trait variation. The fluidity of taxon circumscription demands a dataset-based approach and the TH paradigm provides such platform.

The TH paradigm is not presented as a challenge to current codes of nomenclature, because these deal only with names, not taxon circumscriptions. The paradigm builds on the nomenclature codes, which remain as stable anchors of the taxon communication system. At present, any project that wishes to generate THs could do so, and the TH concept, with the associated PIDs, is being implemented in UNITE. Broadscale integration of the TH paradigm with nomenclature codes could be achieved by introducing compulsory registration of taxon datasets. However, there is a long tradition of nomenclature codes restricting their ambit to names and leaving the regulation of taxonomy to scientific norms such as peer review. Therefore, while there are strict rules, for example, on how to spell a name, there is no restriction in the codes on how a taxonomist should delimit a taxon, although the Prokaryotic Code does recommend minimal standards for character sets [53]. Ultimately, taxon concepts are stabilized through the acceptance by the relevant user community. There are formal procedures for amending codes of nomenclature (either via commissions or voting at international congresses), and it is unlikely that introducing mandatory provisions related to taxonomy would be successful. Indeed, recent discussions on globally agreed name lists have adopted the principle of separating governance of validated lists of species from governance of the naming of species [54].

Nevertheless, registration authorities that are authorized by codes of nomenclature to issue identifiers for names are a logical source of identifiers for taxon hypotheses [55]. A potential scenario is that name repositories introduce the ability to obtain TH identifiers (for appropriately structured data and metadata) and journals encourage their use. There could also be a role in designing and monitoring such a system for the various international commissions under the International Union of Biological Sciences and the International Union of Microbiological Societies, such as the International Commission on the Taxonomy of Fungi. As long as minimum standards of data deposition are adhered to, there is no need to police the issuing of TH identifiers, as whether or not a given TH is adopted is ultimately a matter for the user community.

Issuing of TH identifiers is necessary not only for formally described species (both at description and over time), but also for the species that cannot be formally described under the relevant codes. A very pertinent example, where a species can be hypothesized but not named, is the “dark taxa” that are especially prevalent in microscopic lineages for which environmental sequences are known but specimens have not yet been obtained. It is not possible to create code-compliant names for dark taxa as a type specimen is not available. There are active discussions on the potential formal and informal means of naming dark taxa [56]. Some options include code-compliant naming in the absence of specimens, using DNA sequences as types. Other options take up numbering systems, such as UNITE species hypotheses, as stable means of communicating dark taxa. Although the naming of dark taxa will be dealt with, it is highly likely that we will be dealing with taxa that are recognizable but not formally named for some time, including at ranks higher than species.

Datasets of individuals such as from eDNA samples, which likely represent new species, can be assigned a PID but not a species name. Such datasets may carry a higher-level taxon name if it is possible to place it in the genus, family, order, etc. Datasets connected to identifiers (PIDs) must be machine readable and follow major biological standards. The TH paradigm allows an indefinite number of THs for any individual. The concept of THs also requires that PIDs are attached to the individuals. Only then the system can track the use of the same individuals in different THs. There are a few international bodies or databases that already partly regulate the provision of PIDs to the biological individuals. For example, unique acronyms of the reference collections combined with numbering offers one solution to attach PIDs to the specimens and other individuals lodged in public collections (e.g., WFCC [57], Index Herbariorum [58], and the GBIF registry of scientific collections [9]). Another system was implemented by the International Nucleotide Sequence Database Consortium (INSDC), where individuals and their DNA sequences have globally unique PIDs. The Distributed System of Scientific Collections (DiSSCo [59]) is also making an effort to develop a standard for the Digital Specimens [60]. Their PIDs represent a digitized physical specimen as well as all related information and properties. The UNITE system currently uses all these PID options to make individuals in SHs traceable through the different SH versions and in overall communication of SHs.

The benefits of attaching unique TH identifiers to taxon datasets are: (i) communicating formally described and undescribed species in the same system; (ii) making species and other taxa easily comparable over all studies which are using taxon datasets for the identification and communication; (iii) large biological data portals such as GBIF, which are able to bring together taxon occurrences from different studies and disciplines; (iv) different classifications or taxonomic backbones that are no longer problematic, because THs can be attached to many classifications; and vice versa, a single classification may populate different THs of the same individuals; and (v) higher level taxon names have fixed the content in communication (individuals and their properties), which is largely not the case today.

The UNITE system opened registration of SHs in 2011 by using locally generated PIDs. Starting in 2015, DOIs were used for the registration and communication of SHs. In 2017, UNITE started to register higher-level THs, which were communicated via DOIs from the onset. Datasets and their DOIs are freely downloadable and machine readable for identification service and communication in research papers as well as publication in data portals such as GBIF.

### 4.2. TH Paradigm and Metagenomics

A general obstacle in interpreting and communicating studies based on massive parallel sequencing of genetic markers (DNA metabarcoding) or shotgun sequencing of metagenomes, has been—and still is—a large fraction of terminal taxa (OTUs) that usually cannot be assigned to formally described species. The UNITE and BOLD concepts have greatly benefited the communication of terminal taxa (OTUs) in metabarcoding datasets of fungi and animals, respectively (e.g., [61,62]), by providing a possibility of connecting OTUs to unambiguous SHs or BINs via sequence comparison. For fungi, the UNITE TH system enables the construction of taxon-level traits based on metadata associated with sequence accessions in taxonomic and molecular identification studies [63]. Presently, the SHs of UNITE and the BINs of BOLD approximate species-level taxonomy only, which limits the utility of the information, whereas applying the entire TH concept across taxonomic ranks would expand the taxonomic utility in DNA metabarcoding studies. Firstly, THs enable the communication of the taxonomic composition at any taxonomic level, despite the fact that a majority of taxa cannot be identified at species level. It also enables comparisons of results derived from several different genetic markers potentially having varying levels of conservation and taxonomic bias and across different alternative classifications. Secondly, THs allow the detection and testing of phylogenetic trends in community composition as well as testing evolutionary hypotheses without inferring phylogenies from the investigated sequence data [4]. This is especially valuable for highly variable genetic markers—such as the ITS region for fungi—where global alignment and phylogenetic analyses are not practical or scientifically defensible. The TH concept is also applicable to shotgun metagenome and metatranscriptome data where taxonomic identifications are performed.

## 5. Conclusions

Mycology operates under a nomenclatural code conceived during a time where the study of fungi was largely restricted to physical manifestations of species, and where researchers communicated more or less exclusively through scientific papers. This is no longer an accurate description of mycology, which now deals with a plethora of class- or phylum-level groups known only from DNA sequence data, and where digital communication and representation are the key. Nomenclatural codes are in the process of revision to accommodate these new lines of thought, but the progress is outpaced by advances in high-throughput sequencing methods and computational achievements. We introduce the TH concept to serve as a bridge between the nomenclatural codes and the desire to communicate taxon circumscriptions and recoveries unambiguously also in the absence of formal Latin names. The TH concept does not seek to replace formal Latin names but rather to serve as a vehicle to facilitate and speed up the ulterior goal of erecting Latin names for all extant species and groups of organisms. In the absence of initiatives such as the TH concept, we fear that taxonomy will keep struggling with irreproducible names and taxa of unclear application. This would further devalue taxonomy in a time when taxonomy is needed more than ever.

## Figures and Tables

**Table 1:** Selected examples of UNITE THs. TH and SH codes are hyperlinks to the TH and SH DOI pages.

Classification Level	Taxon Name; TH DOI Code	Taxon Name; TH DOI Code	Taxon Name; TH DOI Code	Taxon Name; TH DOI Code	Taxon Name; TH DOI Code
Kingdom	Fungi; TH005107	Fungi; TH005107	Fungi; TH005107	Amoebozoa; TH005117	Viridiplantae; TH005112
Phylum	Basidiomycota; TH005174	Ascomycota; TH005190	Ascomycota; TH005190	Eumycetozoa; TH005218	Anthophyta; TH005194
Class	Agaricomycetes; TH005482	Lecanoromycetes; TH005556	Eurotiomycetes; TH005377	Dictyostelea; TH005492	Eudicotyledonae; TH005330
Order	Agaricales; TH006249	Lecanorales; TH006023	Eurotiales; TH005852	Dictyosteliida; TH005599	Fagales; TH005787
Family	Schizophyllaceae; TH006801	Parmeliaceae; TH006508	Aspergillacea; TH006460	Dictyosteliidae; TH007268	Fagaceae; TH008199
Species	*Schizophyllum commune*; SH1565276.08FU	*Vulpicida juniperinus*; SH1716443.08FU	*Aspergillus fumigatus*; SH2189906.08FU	*Polysphondylium violaceum*; SH1514152.08FU	*Quercus suber*; SH1599838.08FU

**Table 2:** Examples of the formally described species which were communicable via UNITE SHs before publication.

Species, Publication Year	UNITE SH; the First Publication Year	Reference
*Bifiguratus adelaidae* 2017	https://unite.ut.ee/bl_forw_sh.php?sh_name=SH022292.06FU#fndtn-panel1; 2013	[43]
*Cortinarius koldingensis* 2015	https://unite.ut.ee/bl_forw_sh.php?sh_name=SH201833.06FU#fndtn-panel1; 2013	[42]
*Polyozellus atrolazulinus* 2018	https://unite.ut.ee/bl_forw_sh.php?sh_name=SH028342.07FU#fndtn-panel1; 2014	[44]

## Appendix A

### Appendix A.1. Taxon Hypothesis

A taxon hypothesis (TH) is a digital dataset of individuals and their properties (traits/characters) with associated metadata. Every TH may include an indefinite number of individuals and their traits. Each TH is published with a persistent identifier (PID) which enables precise communication of a dataset with a fixed number of individuals and traits. The PID furthermore enables the linking of different versions of the datasets across time. THs are placed in taxonomic classification if individuals are identified at a species or higher level. When the identification or classification of a TH changes, the PID-based THs maintain the communication between different taxonomic backbones (a predefined hierarchical taxonomic system). When new individuals or traits are added to a TH, a new unique and stable PID is provided and a new TH is formed. In this way, the ID of the TH always refers to a static dataset. Individuals also have unique PIDs, which makes it possible to track them through the different versions of THs and across other datasets. Traits and metadata of the individuals are atomized and stored in TH datasets according to the fixed standards. Therefore, THs are machine readable and can be automatically constructed, compared, and used in identification and communication processes. Most importantly, such THs can be repeatedly verified or falsified in an automated way.

### Appendix A.2. Species Hypothesis Is a Distinct Case of the Taxon Hypothesis

Species is the principal taxon identified and communicated in science and in the media. Therefore, our paper focuses on species hypotheses (SH) when describing the founding principles of the TH paradigm. However, SH is just an example of a TH that is formed by individuals and their characters or traits, just like THs at other classification levels.

### Appendix A.3. Individuals

In the context of this paper, a biological individual can be any part of the organism or the full organism. Examples of individuals include a single cell or its contents, such as organelles, or even purified DNA. An individual can also be a full organism, such as a fungal mycelium in the soil, a single cat, or a single plant with below- and aboveground parts. The organisms living inside other organisms or forming complex organisms with others are separate individuals. The most important feature of the individual is that it must display properties which enable its identification and placement in a biological communication system.

A digital individual presents a case where a physical individual and its properties are processed digitally. A digital individual may have several instances distributed in different databases. For example, data on specimens may reside in a collection database, but the corresponding DNA sequences are stored in a sequence database.
